# Dietary Influence on Bladder Pain Syndrome: A Systematic Review

**DOI:** 10.7759/cureus.69437

**Published:** 2024-09-15

**Authors:** Sulaiman Almutairi

**Affiliations:** 1 Department of Urology, College of Medicine, Majmaah University, Al-Majmaah, SAU

**Keywords:** bladder pain syndrome, diet, dietary influence, nutritional factors, systematic review, urinary symptoms

## Abstract

Bladder Pain Syndrome (BPS) is a chronic condition characterized by discomfort or pain in the bladder region, often exacerbated by bladder filling and alleviated by voiding. Despite numerous theories regarding its etiology, the potential dietary influence on BPS symptoms has not been thoroughly elucidated. This systematic review aimed to synthesize evidence on the relationship between nutritional factors and the exacerbation or amelioration of BPS symptoms.

An extensive search was conducted across multiple electronic databases, including PubMed/MEDLINE, EMBASE, Cochrane Library, Web of Science, Scopus, CINAHL, and Google Scholar, to identify studies exploring the impact of diet on interstitial cystitis/bladder pain syndrome(IC/BPS). Comparative analysis was employed to synthesize data from the selected studies, focusing on identifying corroborative and conflicting evidence regarding diet and IC/BPS.

The analysis revealed recurring themes across the eight selected studies, including the association of certain foods and beverages with the worsening of IC/BPS symptoms. Patients frequently reported dietary sensitivities, particularly to acidic and spicy foods, caffeine, and alcohol. Evidence from the studies suggests that dietary modifications, both self-directed and structured interventions, may improve symptom severity and overall patient quality of life. Additionally, tools developed and validated for assessing dietary sensitivities could facilitate better management of IC/BPS through personalized diet plans. The impact of individual substances such as caffeine and tea was underscored, indicating their potential as modifiable risk factors in IC/BPS symptomatology.

The collective evidence from the reviewed studies confirms the importance of dietary influence on IC/BPS symptom management. An individualized approach to dietary counseling based on patient sensitivities could be beneficial. However, the diversity in study methodologies and outcomes indicates a need for more uniform research to establish standardized dietary guidelines for IC/BPS patients.

## Introduction and background

Bladder Pain Syndrome (BPS), also known as interstitial cystitis (IC), represents a significant clinical challenge due to its enigmatic nature and profound impact on quality of life [1]. Patients with BPS frequently report chronic pelvic discomfort, urinary urgency, and frequency, which are often exacerbated by certain lifestyle factors. Among these factors, diet has been frequently implicated by both patients and healthcare providers as a potential modulator of symptom severity [2].

The human diet, rich in variety and complexity, can influence a range of physiological processes, and there is a growing body of literature suggesting that it may have a direct or indirect impact on the symptoms experienced by BPS sufferers. Researchers hypothesize that the acidic or alkaline nature of foods, certain food chemicals, and overall dietary patterns can affect the bladder lining and urinary milieu, potentially aggravating the condition [3]. The modern healthcare framework increasingly recognizes the integration of dietary interventions as a critical element in managing BPS [2]. Consequently, a myriad of clinical protocols endorsed by associations specializing in urology, urogynecology, and gynecology now routinely feature guidance on dietary and lifestyle modifications for the management of urinary tract disorders, reflecting a broad consensus on this issue [3-7].

These disorders are symptomatic manifestations that can occur during various functional phases of the bladder, namely the storage, voiding, and post-micturition phases [8]. The storage phase is characterized by the gradual accommodation of urine in a compliant bladder. Dysfunction during this phase often involves involuntary detrusor muscle contractions, which may be spontaneous or induced [9]. The array of symptoms associated with storage dysfunction encompasses conditions such as urinary urgency, which is a compelling need to urinate that is frequently experienced during the day; urinary frequency, defined as an increased daytime need to void; urinary incontinence, which involves the unintentional loss of urine; nocturia, necessitating awakening at night one or more times to urinate; and discomfort or pain during the bladder filling process [10-11].

Although dietary recommendations are widely used in BPS management, there is a lack of robust evidence to support specific dietary interventions. The potentially pivotal role of diet is still debatable, with conclusions often drawn from anecdotal evidence or studies with limited scope. Thus, a need exists to consolidate research findings to understand the role diet may play in the onset, progression, or alleviation of BPS symptoms. To address this gap, this systematic review aims to synthesize current evidence regarding the dietary influence on bladder pain syndrome. We will examine the relationship between dietary intake and BPS symptomatology, evaluate the quality of the studies conducted in this area, and quantify the effects of specific dietary components on patient outcomes. In doing so, we hope to provide a clearer understanding of the role of diet in managing BPS and offer guidance for clinicians and patients alike in making informed dietary choices to alleviate the burden of this challenging condition.

## Review

Methodology

In conducting this review, the PRISMA (Preferred Reporting Items for Systematic Reviews and Meta-Analyses) guidelines [11] were adhered to, ensuring a rigorous and transparent approach to synthesizing the existing literature on the relationship between diet and IC/BPS. Initially, an a priori protocol was developed outlining the objectives, criteria for study inclusion and exclusion, and intended analysis methods. This protocol served as a blueprint throughout the review process. The PECO framework utilized in this review is as follows:

Population: Individuals diagnosed with BPS/IC.

Exposure: Consumption of specific dietary components or adherence to particular nutritional patterns.

Comparator: Absence of dietary exposure, or comparison between different types or levels of dietary exposure, although the comparator group was not necessarily mandatory, considering the objectives of this review.

Outcome: The prevalence or severity of bladder pain syndrome symptoms, changes in urinary frequency, pain reduction, and quality of life improvements.

Table 1 delineates the different inclusion and exclusion criteria that were utilised for the review.

**Table 1:** Inclusion and exclusion criteria IC/BPS: Interstitial cystitis/bladder pain syndrome

Criteria	Inclusion	Exclusion
Population	Adults diagnosed with IC/BPS according to accepted clinical criteria.	Studies including patients with other primary urological conditions.
Intervention	Studies that assessed dietary interventions, modifications, or dietary patterns.	Studies focusing on pharmacological, surgical, or non-dietary interventions alone.
Comparator	Any comparator, including different types of dietary interventions, usual care, or no dietary intervention.	-
Outcomes	Measures of IC/BPS symptoms, quality of life, urinary markers, or other relevant clinical outcomes.	Studies reporting unrelated outcomes, such as unrelated urological or gynecological outcomes.
Study Design	RCTs, cohort studies, case-control studies, cross-sectional studies.	Case reports, editorials, opinion pieces, reviews without original data, animal studies.
Publication	Peer-reviewed articles published in journals.	Grey literature, abstracts, conference proceedings, unpublished data, theses and dissertations.

Database Search Protocol

For this systematic review, a comprehensive and structured search strategy was developed and implemented across multiple databases, including PubMed/MEDLINE, EMBASE, Cochrane Library, Web of Science, Scopus, CINAHL, and Google Scholar. The search protocol was devised to harness both text words and controlled vocabulary, such as MeSH (Medical Subject Headings) terms, to increase the sensitivity of the search, the results of which have been displayed in Table 2:

**Table 2:** Search strings deployed across the different assessed databases IC/BPS: Interstitial cystitis/bladder pain syndrome

Database	Search String
PubMed/MEDLINE	("Interstitial Cystitis"[MeSH Terms] OR "Bladder Pain Syndrome"[All Fields] OR IC/BPS[All Fields]) AND ("Diet"[MeSH Terms] OR "Diet Therapy"[MeSH Terms] OR "Dietary Habits"[All Fields] OR "Food and Beverages"[All Fields] OR "Nutritional Status"[MeSH Terms] OR "Diet Records"[All Fields] OR "Food Hypersensitivity"[MeSH Terms] OR "Diet Surveys"[MeSH Terms])
EMBASE	('interstitial cystitis'/exp OR 'bladder pain syndrome':ab,ti OR 'IC/BPS':ab,ti) AND ('diet'/exp OR 'diet therapy'/exp OR 'dietary habit':ab,ti OR 'food and beverages':ab,ti OR 'nutritional status'/exp OR 'diet record':ab,ti OR 'food hypersensitivity'/exp OR 'diet survey':ab,ti)
Cochrane Library	(MeSH descriptor: [Interstitial Cystitis] OR "Bladder Pain Syndrome" OR IC/BPS) AND (MeSH descriptor: [Diet] OR "Diet Therapy" OR "Dietary Habits" OR "Food and Beverages" OR "Nutritional Status" OR "Diet Records" OR "Food Hypersensitivity" OR "Diet Surveys")
Web of Science	(TS=("Interstitial Cystitis" OR "Bladder Pain Syndrome" OR IC/BPS)) AND (TS=("Diet" OR "Diet Therapy" OR "Dietary Habits" OR "Food and Beverages" OR "Nutritional Status" OR "Diet Records" OR "Food Hypersensitivity" OR "Diet Surveys"))
Scopus	(TITLE-ABS-KEY("interstitial cystitis") OR TITLE-ABS-KEY("bladder pain syndrome") OR TITLE-ABS-KEY(ic/bps)) AND (TITLE-ABS-KEY(diet) OR TITLE-ABS-KEY("diet therapy") OR TITLE-ABS-KEY("dietary habits") OR TITLE-ABS-KEY("food and beverages") OR TITLE-ABS-KEY("nutritional status") OR TITLE-ABS-KEY("diet records") OR TITLE-ABS-KEY("food hypersensitivity") OR TITLE-ABS-KEY("diet surveys"))
CINAHL	(MH "Interstitial Cystitis" OR "Bladder Pain Syndrome" OR IC/BPS) AND (MH "Diet+" OR "Diet Therapy" OR "Dietary Habits" OR "Food and Beverages" OR "Nutritional Status" OR "Diet Records" OR "Food Hypersensitivity" OR "Diet Surveys")
Google Scholar	("Interstitial Cystitis" OR "Bladder Pain Syndrome" OR IC/BPS) AND ("Diet" OR "Diet Therapy" OR "Dietary Habits" OR "Food and Beverages" OR "Nutritional Status" OR "Diet Records" OR "Food Hypersensitivity" OR "Diet Surveys")

Data Selection Process

The data extraction protocol for the systematic review was meticulously planned to ensure systematic and consistent capture of all relevant information from the included studies. A standardized data extraction form was developed and pilot-tested on a subset of included studies to refine the data collection process and ensure its comprehensiveness. Two independent reviewers conducted the data extraction, and discrepancies were resolved through discussion or involving a third reviewer when necessary.

The data items selected for extraction were determined based on their relevance to the review objectives and their potential to contribute to the synthesis of findings. The following data items were included:

Study Characteristics: Detailed information regarding the study design (e.g., RCT, cohort, case-control, cross-sectional) and setting were recorded.

Participants: Data were extracted on the number of participants, participant demographics (age, sex, ethnicity), diagnostic criteria used for IC/BPS, and baseline characteristics.

Interventions and Comparators: For each study, the specifics of the dietary intervention (type, duration, and nature of the dietary advice or regimen) and the comparator (if applicable) were documented.

Outcomes: The primary and secondary outcomes measured were recorded, including the instruments or methods used for assessment (e.g., questionnaires, biomarkers), the time points at which outcomes were measured, and any follow-up data.

Results: Key findings related to the review’s objectives, including effect sizes, statistical significance, and confidence intervals, were extracted.

Risk of Bias: Information pertinent to assessing the risk of bias in individual studies was collected based on the tool appropriate to the study design (e.g., the Cochrane risk of bias tool for RCTs).

Bias Assessment Tools

The bias assessment protocol was constructed to ensure a thorough and objective evaluation of the risk of bias in the included studies. The protocol utilized a tripartite approach, incorporating the Cochrane Risk of Bias 2.0 tool [12] (Rob 2.0, Cochrane Methods, London, UK) for RCTs, the AXIS (Appraisal Tool for Cross-Sectional Studies) tool [13], and the Risk of Bias in Non-randomized Studies-of Interventions (ROBINS-I) tool (Cochrane Methods, London, UK) [14] for non-randomized studies.

Certainty of Evidence

Upon completion of the bias assessment using the Cochrane RoB 2.0 [12], AXIS [13], and ROBINS-I tools [14], the certainty of the evidence for each outcome across the studies was evaluated using the Grading of Recommendations Assessment, Development, and Evaluation (GRADE) approach [15]. The GRADE framework allows the rating of the quality of evidence and strength of recommendations, considering study limitations, inconsistency of results, indirectness of evidence, imprecision, and publication bias.

Each outcome from the included studies underwent a process where the initial level of evidence was considered high if sourced from RCTs or moderate if from observational studies. From there, the quality of the evidence was downgraded by one or two levels if severe limitations were detected, respectively.

Results

Study Selection Process

**Figure 1:**
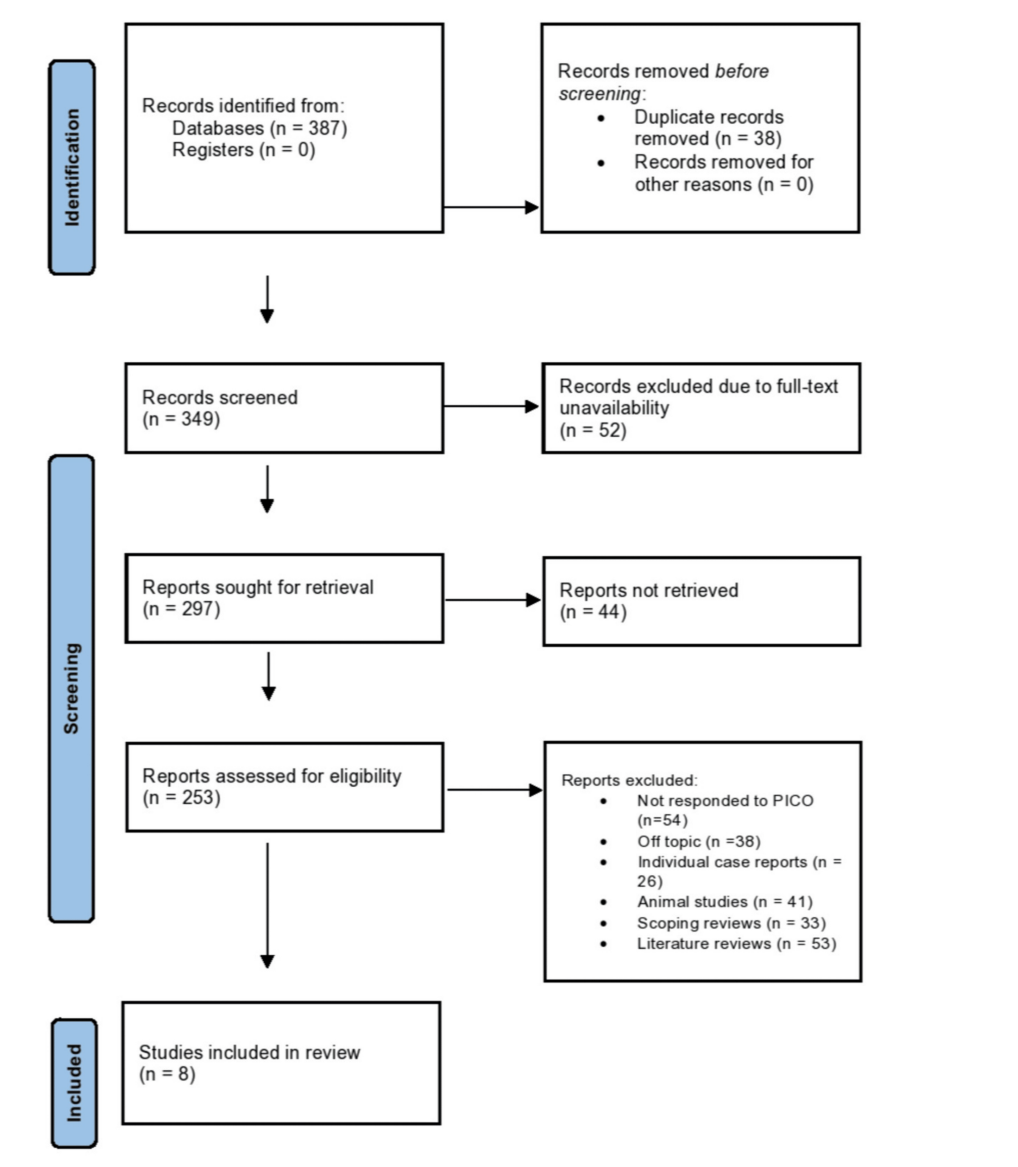
Article selection process representation of the review

As elucidated in Figure 1, the article selection process for the review commenced with identifying records from databases, which amounted to 387 entries. There were no records identified in the registers. Before the screening process, 38 duplicate records were removed. No records were removed for other reasons at this stage. This left 349 records for screening. Upon screening, 52 records were excluded due to the unavailability of the complete text, reducing the number of records further to 297 that were sought for retrieval. However, 44 were not retrieved, meaning 253 reports were assessed for eligibility. In the eligibility assessment phase, several reports were excluded based on specific criteria: 54 were excluded for not responding to the PICO framework; 38 were considered off-topic; individual case reports accounted for 26 exclusions; 41 were animal studies not relevant to the review; 33 were scoping reviews; and 53 were literature reviews. After applying these exclusion criteria, only eight studies [16-23] remained that were included in the review.

Bias Selection Observations

**Figure 2:**
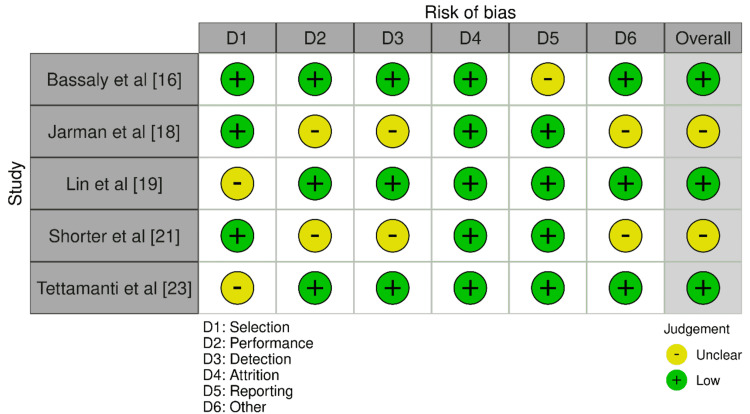
Bias assessment using the AXIS tool AXIS: Appraisal Tool for Cross-Sectional Studies

The AXIS tool (Figure 2) predominantly assessed the study by Bassaly et al. [16] as having a low risk of bias across most domains, including selection, performance, detection, attrition, and other biases. However, the study did face a moderate risk in the reporting domain, pointing to potential issues with the comprehensiveness of reporting study procedures or findings. The studies by Jarman et al. [18] and Shorter et al. [21] showed low risk in selection and detection. Still, they exhibited moderate risk in performance and other biases, resulting in a moderate overall bias risk. This suggests that while participant selection and outcome measurement were generally unbiased, there were concerns with how participants were managed or unspecified factors that might influence the outcomes. Lin et al. [19] and Tettamanti et al. [23] demonstrated a moderate risk in selection but a low risk in all other domains, resulting in a low overall risk of bias. These findings highlight concerns related to participant selection despite the otherwise sound methodology.

**Figure 3:**
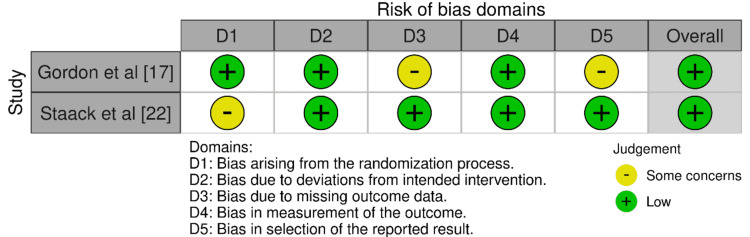
Bias assessment using the RoB 2.0 tool RoB2.0: Cochrane Risk of Bias 2.0 tool for RCTs [13].

**Figure 4:**
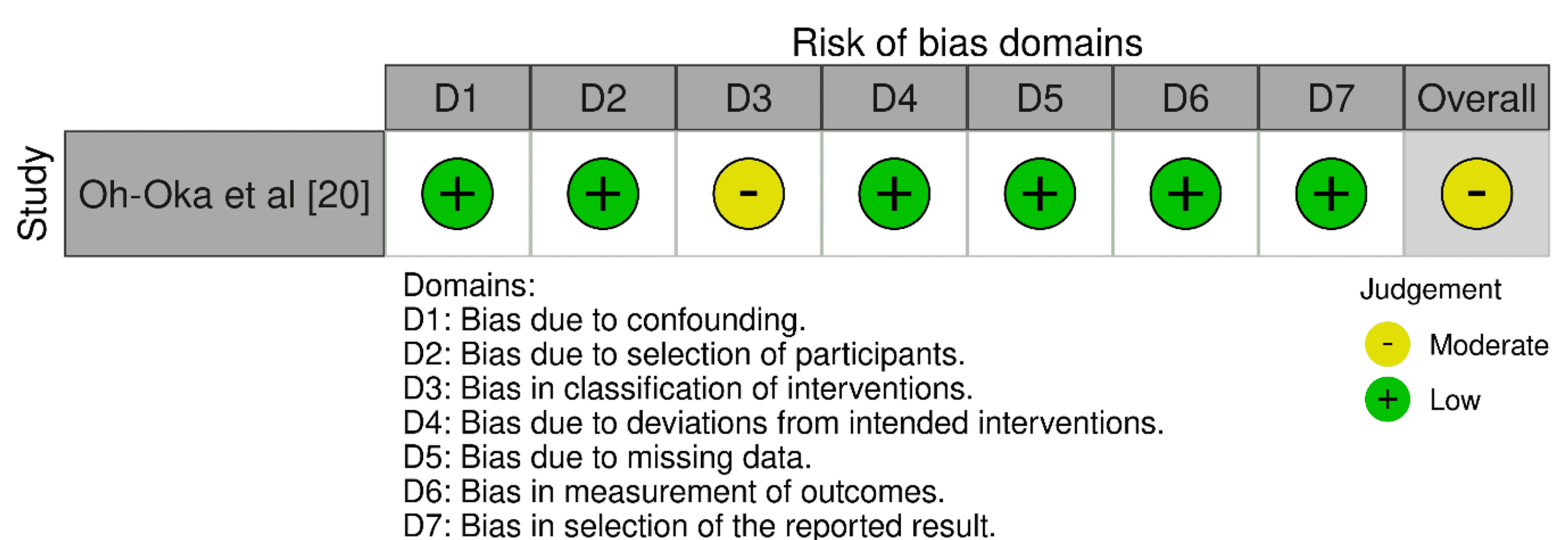
Bias assessment using the ROBINS-I tool ROBINS-I: Risk of Bias in Non-randomized Studies-of Interventions (ROBINS-I) tool (Cochrane Methods, London, UK) for non-randomized studies [14].

In the RoB 2.0 tool assessment (Figure 3), the randomized trial by Gordon et al. [17] scored low in most domains but had some concerns in domains related to participant deviations from intended interventions (D3) and the selection of the reported result (D5). The study maintained a low overall risk of bias despite these concerns. Staack et al. [22] doubted the randomization process (D1) but maintained a low risk in all other domains, ensuring its overall bias remained low. Using the ROBINS-I tool for non-randomized studies (Figure 4), Oh-Oka et al. [20] found a low risk of bias in most domains except for a moderate risk in the domain concerning the selection of participants in the study (D3), leading to a moderate overall risk of bias.

Study Protocols and Cohorts Explored

Table 3 elucidates the included papers and their associated assessments.

**Table 3:** Studies included in the review and their observed inferences IC/BPS: Interstitial cystitis/bladder pain syndrome

Author ID	Design	Cohort description	Evaluated cohorts	Principal discoveries	Summary of findings
Bassaly et al [16]	Cross-sectional	IC/BPS affected members of the Interstitial Cystitis Association	Participants of the survey	High percentage (95.8%) reported exacerbation of IC/BPS symptoms from certain foods and drinks. Specific items, especially acidic, spicy, and carbonated ones, worsened symptoms. Calcium glycerophosphate and sodium bicarbonate were exceptions, showing potential symptom improvement.	The survey indicates a strong correlation between IC/BPS symptom aggravation and consumption of certain foods and beverages, highlighting the potential benefit of dietary modifications.
Gordon et al [17]	RCT	Women with IC/BPS	Women assigned to plant-based, low saturated fat diet (Anti-Inflammatory Diet for Interstitial Cystitis - AID-IC) vs. control diet	Significant reductions in solid fats, saturated fat, and refined grains intake on AID-IC diet; increased seafood and vitamin B12 intake; better quality of life indicators in the treatment group; no significant changes in other nutrients or empirical dietary inflammatory index (E-DII) scores when comparing diets	The AID-IC diet appears to lead to a healthier dietary pattern and may improve certain quality of life aspects for women with IC/BPS, although no significant association with disease activity was found based on E-DII scores.
Jarman et al [18]	Cross-sectional	Veterans with ICD-9/10 codes for IC/BPS	IC/BPS patients, other pelvic pain (OPP), healthy controls (HC)	IC/BPS patients reported more food sensitivities, especially to acidic, spicy foods, and certain beverages; Black IC/BPS patients had more sensitivities and worsened urgency than White patients	Food sensitivities are notably higher in IC/BPS patients, indicating the Shorter Moldwin Food Sensitivity Questionnaire (SMQ) could be a useful tool for distinguishing IC/BPS from other pelvic pain conditions.
Lin et al [19]	Cross-sectional	Patients with suprapubic pain, lower urinary tract symptoms, and cystoscopic findings	IC/BPS patients and age-matched controls without IC/BPS	IC/BPS patients reported higher cranberry intake and altered lifestyle behaviors like reduced coffee and spicy food consumption, and less use of makeup or special work garments	IC/BPS patients appear to adapt their lifestyle and dietary habits in response to their condition, potentially to alleviate symptoms.
Oh-Oka et al [20]	Interventional study with nutritional guidance	IC/BPS patients undergoing dietary intervention	Intensive Dietary Management (ISDM) group, Non-Intensive Dietary Management (NIDM) group	All measured factors (symptom indices, urgency score, pain score, quality of life) improved significantly in the ISDM group over 3 months and 1 year; no significant improvements in NIDM group	The ISDM approach, avoiding certain food items, significantly improved symptoms and quality of life in IC/BPS patients over the long term, with effects persisting for at least a year.
Shorter et al [21]	Cross-sectional	Patients with self-reported food sensitivities	124 patients initially; 52 completed both surveys	High internal consistency (α = 0.96) for the questionnaire; 90.4% reported food sensitivity; test-retest reliability ranged from moderate to very strong	The abbreviated survey demonstrated strong reliability and consistency, showing that most patients with bladder pain syndrome report food sensitivities.
Staack et al [22]	RCT	Subjects with dietary caffeine restriction	Subjects assigned to regular coffee (high caffeine) or decaffeinated coffee (low caffeine)	Subjects consuming high-caffeine coffee reported increased urgency and frequency; low-caffeine users showed the greatest symptom increase; no significant change in urinary symptoms with decaffeinated coffee	Avoiding high-caffeine coffee can prevent urinary urgency and frequency, suggesting a dietary influence on bladder pain syndrome symptoms.
Tettamanti et al [23]	Cross-sectional	Female twins in Sweden with information on bladder pain	Female twins with BPS symptoms	Tea consumption associated with increased risk of BPS; smoking linked to higher risk but may be confounded by familial factors; coffee not a significant risk factor	Tea consumption may increase the risk of BPS, while smoking's association with BPS could be influenced by genetic or family-related factors.

Bassaly et al. [16] conducted a cross-sectional study targeting members of the Interstitial Cystitis Association. The study surveyed these individuals to understand the prevalence of IC/BPS in this population. Gordon et al. [17] implemented an RCT with women diagnosed with IC/BPS. Gordon et al. [17] divided the participants into two groups: one that followed a plant-based, low-saturated-fat diet known as the Anti-Inflammatory Diet for Interstitial Cystitis (AID-IC) and a control group that adhered to their usual diet. The outcomes assessed the impact of the AID-IC diet on IC/BPS symptoms and quality of life. Jarman et al. [18] conducted another cross-sectional study comparing Veterans Affairs patients with IC/BPS, identified through ICD-9/10 codes, with patients who presented with OPP and a healthy HC. This study aimed to determine the prevalence of food sensitivities reported by patients with IC/BPS compared to other groups.

Lin et al. [19] also utilized a cross-sectional design to compare patients with IC/BPS, characterized by suprapubic pain, lower urinary tract symptoms, and cystoscopic findings, against age-matched controls without IC/BPS. The study assessed lifestyle adjustments, including dietary habits, that patients with IC/BPS may adopt in response to their symptoms. Oh-Oka et al. [20] conducted an interventional study providing nutritional guidance to IC/BPS patients. They divided the participants into two groups: an intensive diet management (IDM) group and a non-intensive diet management (NIDM) group. The study aimed to evaluate the effects of dietary modifications on IC/BPS symptomatology.

Shorter et al. [21] assessed food sensitivities in patients with self-reported sensitivities using a cross-sectional approach. Only 52 of the initial 124 patients completed the initial and follow-up surveys. The study provided insights into the prevalence and nature of food sensitivities in the patient population. In an RCT, Staack et al. [22] assigned subjects with dietary caffeine restrictions to either consume regular coffee (high caffeine) or decaffeinated coffee (low caffeine). This study aimed to evaluate the impact of caffeine consumption on IC/BPS symptoms. Tettamanti et al. [23] conducted a cross-sectional survey of female twins in Sweden to explore the environmental and genetic influences on BPS. The study included twins with BPS symptoms, and logistic regression was used to estimate BPS risk factors. The study aimed to determine the heritability of BPS and the extent to which genetic and environmental factors contribute to the development of the condition.

Principal Findings

Bassaly et al. [16] found that a striking majority (95.8%) of their surveyed cohort reported exacerbations in IC/BPS symptoms following the consumption of particular foods and beverages. Notably, acidic, spicy, and carbonated foods were implicated in worsening symptoms. Researchers also identified substances like calcium glycerophosphate and sodium bicarbonate as potential mitigators of symptoms. Gordon et al. [17] reported that subjects adhering to the AID-IC diet experienced significant dietary changes, specifically a reduction in solid fats, saturated fats, and refined grains, coupled with an increase in seafood and vitamin B12 intake. Crucially, the treatment group exhibited enhanced quality of life indicators despite the absence of significant changes in other nutrients or empirical dietary inflammatory index (E-DII) scores when comparing the various diets.

Jarman et al. [18] observed that IC/BPS patients had heightened food sensitivities, particularly aversions to acidic and spicy foods and certain beverages. An intriguing demographic finding was that Black patients with IC/BPS reported more sensitivities and an increased sense of urgency compared to their White counterparts. Lin et al. [19] noted that IC/BPS patients had a higher intake of cranberries and had altered various lifestyle behaviors, presumably to mitigate symptoms. These changes included reducing coffee and spicy food consumption, as well as modifications in cosmetic products and work attire.

The interventional study by Oh-Oka et al. [20] demonstrated significant improvements across various parameters, including symptom indices, urgency, pain scores, and quality of life measures, in the IDM group over periods of 3 months and one year. Interestingly, the Non-Intensive Dietary Management (NIDM) group did not replicate these improvements. Shorter et al. [21] validated their questionnaire with high internal consistency (Cronbach’s alpha = 0.96) and found that 90.4% of respondents reported food sensitivities. The test-retest reliability of the questionnaire ranged from moderate to very strong, emphasizing the robustness of the instrument in capturing patients’ dietary sensitivities.

Staack et al. [22] provided insights into the effects of caffeine on IC/BPS symptoms. Individuals consuming high-caffeine coffee reported an increase in urinary urgency and frequency. Low-caffeine coffee consumers observed the most substantial symptom exacerbation, suggesting a possible nocebo effect or other confounding variables affecting their symptoms. Tettamanti et al. [23] identified tea consumption as a potential risk factor for BPS. Furthermore, the study on twins suggested that familial or genetic predispositions might confound the association between smoking and a higher risk.

GRADE Assessment Observations

Interpreting the findings from Table 4, the high to moderate level of confidence in the evidence reflects a general agreement among the studies regarding the impact of dietary factors on IC/BPS despite variations in specific outcomes and dietary components assessed. The collective evidence from the five cross-sectional studies [16, 18-19, 21, 23] contributes to a consistent narrative that diet and food sensitivities play a role in the symptoms experienced by IC/BPS patients. Given their observational nature, these studies provide less robust evidence than randomized controlled trials.

**Table 4:** GRADE assessment observation pertaining to publication bias in the included studies IC/BPS: Interstitial cystitis/bladder pain syndrome; AID-IC: Anti-Inflammatory Diet for Interstitial Cystitis

Research Methodology	Number of studies	Common Outcome	Bias Assessment	Consistency of Findings	Directness of Evidence	Accuracy of Data	Additional considerations	Level of confidence
Cross-sectional Studies	5	Correlation between diet and IC/BPS symptoms, higher food sensitivities, lifestyle adaptation, reliability of survey on food sensitivities, tea consumption risk	Low	Not applicable	High	Moderate	None	High
RCTs	2	Improved quality of life with AID-IC diet, reduction of urinary urgency with low-caffeine diet	Low to moderate	Not applicable	High	Moderate	None	Moderate
Interventional Study with Nutritional Guidance	1	Long-term symptom improvement	Low to moderate	Not applicable	High	Moderate	None	Moderate

The two RCTs [17, 22] provided a more reliable form of evidence with a low to moderate risk of bias. Still, the nature of the outcomes and the interventions varied, making it difficult to aggregate the findings into a consistent effect. Despite this, the RCTs add valuable insights into the potential benefits of dietary modifications such as the AID-IC diet and low caffeine consumption, with both studies indicating positive outcomes for IC/BPS patients.

The single interventional study [20] with nutritional guidance also supported the proposition that diet has a therapeutic role in managing IC/BPS symptoms, aligning with the common finding of the potential benefit of dietary modifications. However, the evidence from a single study must be interpreted with caution.

Discussion 

A nuanced narrative regarding the relationship between diet and IC/BPS emerges from a thematic review of the included papers, revealing both convergences and divergences in the scholarly discourse. Bassaly et al. [16] and Jarman et al. [18] provide complementary findings regarding the dietary sensitivities experienced by IC/BPS patients. Both studies supported the idea that certain foods and drinks worsen IC/BPS symptoms. Jarman et al. [18] also found that IC/BPS patients were likelier than controls to report food allergies. These studies collectively underscore the necessity for dietary considerations in IC/BPS management.

Gordon et al. [17] and Oh-Oka et al. [20] both delved into dietary modifications as a means of improving IC/BPS symptoms. Gordon et al. [17] focused on adopting the AID-IC diet and its positive impact on quality of life. Oh-Oka et al. [20] demonstrated significant symptomatic and quality-of-life improvements persisting over a year through the ISDM approach. According to the E-DII scores, Gordon et al. [17] found no significant link between dietary changes and disease activity. However, the positive quality of life outcomes is similar to those found by Oh-Oka et al. [20], which suggests that dietary interventions, even if different in their mechanisms, may help with symptoms.

Lin et al. [19] shared a similar theme with the studies above regarding lifestyle and dietary modifications adopted by IC/BPS patients. However, instead of focusing on structured dietary interventions, the study highlighted the spontaneous nutritional changes made by patients, such as increased cranberry intake and decreased coffee consumption. This behavioral adaptation supports the broader inference that IC/BPS patients adjust their diets in response to their symptoms. The survey by Shorter et al. [21] reinforced the recurring theme of food sensitivity among IC/BPS patients, showing alignment with the findings of Bassaly et al. [16] and Jarman et al. [18]. However, Shorter et al. [21] added dimension by verifying the reliability and consistency of the survey tool used to measure food sensitivities, thereby strengthening the methodological underpinnings of dietary research in IC/BPS.

Staack et al. [22] and Lin et al. [19] both observed the impact of caffeine on IC/BPS symptoms, with Staack et al. [22] offering a more detailed examination of the effects of varying caffeine levels in coffee. Although the studies differed in specificity, they jointly suggest caffeine may be a modifiable dietary factor for IC/BPS symptom management. Tettamanti et al. [23] diverged from the other studies by identifying tea consumption, rather than just acidic or spicy foods, as a potential risk factor for BPS. While not directly related to diet, the association with smoking introduces an additional lifestyle factor into the conversation, albeit with the caveat of potential genetic confounders.

Dietary modification is a critical strategy in managing bladder storage dysfunctions, focusing on reducing the intake of substances identified as bladder irritants [24-25]. These irritants commonly include alcohol, caffeine, citrus juices, carbonated drinks, and spicy foods. Despite the prevalence of such guidelines, there is a notable lack of robust clinical evidence to substantiate the impact of dietary choices on bladder storage disorders. Most recommendations are derived from fundamental research and observational cohorts, with only a limited number of dietary guidelines supported by high-quality evidence [26-28]. Specifically, six out of eight well-supported recommendations pertain to dietary counseling targeted at obesity reduction to alleviate symptoms of overactive bladder (OAB) and stress urinary incontinence (SUI). Another recommendation endorses limiting fluid intake in the evening for individuals with OAB, while the last advises dietary counseling for concurrent conditions in patients with IC/BPS [26-28]. Furthermore, there is a noticeable lack of consensus among experts on the potential efficacy of nutritional interventions for urological conditions, emphasizing the need for standardized dietary guidelines.

Foods that irritate the bladder have been studied in both animal models and in vitro settings before being tested on people. The results show that these foods can affect bladder function and help cause urinary disorders. Artificial sweeteners, such as acesulfame K, aspartame, and sodium saccharin, have been implicated in stimulating bladder activity [29, 30]. Research by Elliot and colleagues suggests that the presence of T1R2/3 sweet taste receptors in the bladder urothelium could be associated with detrusor muscle contractions [29]. Dasgupta and his colleagues showed that even small amounts of artificial sweeteners can enhance detrusor contractions by altering L-type Ca^2+ channels [30]. Studies have linked caffeine to increased bladder instability, urinary frequency, overactivity of the bladder detrusor, and changes in the bladder epithelium, all critical for muscle contraction [31]. The physiological impact of caffeine, even at low doses, may contribute to the urgency of urination. Avelino and Cruz have identified caffeine’s interaction with capsaicin-sensitive ion channels, which are involved in pain perception and bladder contractions, potentially elucidating the mechanism by which caffeine intake may trigger symptoms in IC/BPS [32]. Citrus products contain ascorbic acid, which is associated with increased frequency and intensity of bladder muscle contractions, possibly through the modulation of neurotransmitter activity [33]. Furthermore, studies have observed that the components of carbonated sodas, including ascorbic acid, citric acid, phenylalanine, and colorants, enhance detrusor contractions [34]. Spicy foods, through their activation of transient receptor potential (TRP) channels in sensory nerve endings, can cause irritation and inflammation, which may intensify bladder pain [28, 34].

The review by Verghese et al. [35] assessed the effectiveness of various complementary therapies, including dietary management, for BPS. Their conclusion that dietary management could benefit patients aligns with our review’s findings, which also emphasize the role of diet in managing interstitial cystitis/bladder pain syndrome (IC/BPS). Both reviews acknowledged the potential for dietary intervention to alleviate symptoms. However, Verghese et al. [35] derived their conclusions from a mix of RCTs and prospective studies, noting the small size of these studies and the need for more robust trials. This cautionary note is a joint scientific stance when affirming treatment efficacy, especially when existing studies are limited in scale and scope.

Gordon B. [36] compared clinical guidelines for urological conditions and found that nutrition recommendations were most extensive for IC/BPS. This finding complements our review’s collective inference that individualized dietary interventions may enhance IC/BPS management. The emphasis on nutrition in the guidelines supports our review’s identification of diet as a significant factor. However, Gordon B. [36] also highlighted the variability in dietary manipulation recommendations for different urological conditions and the importance of clinician awareness of evidence limitations. Their study underscored the need for medical nutrition therapy, which suggests that while our review focused on the impact of diet, it is also critical to consider the broader context of nutrition-related comorbidities and the professional guidance required for effective dietary management.

Friedlander et al. [37] reported high percentages of IC/BPS patients experiencing food sensitivities, listing specific foods that exacerbate or alleviate symptoms. These findings are consistent with our review’s results, where certain foods and beverages were identified as exacerbating IC/BPS symptoms. Both reviews suggest that an elimination diet can be a controlled method to determine individual sensitivities, essential for maintaining optimal nutrition while managing symptoms. Friedlander et al. [37] also discussed the pathophysiological mechanisms linking diet and IC/BPS, such as neural upregulation and bladder epithelial dysfunction, which were not explicitly detailed in our review. Their focus on questionnaire-based data and the impact of comorbid conditions provides a complementary perspective to our review’s emphasis on dietary modifications and the patient’s spontaneous dietary changes in response to symptoms.

Bradley et al. [38] provided a systematic literature review that considered a broad range of nonurological factors, including diet, fluid intake, caffeine, alcohol, and tobacco use, and their effects on lower urinary tract symptoms. They found that increased fluid and caffeine intake were associated with urinary frequency and urgency. At the same time, modest alcohol use seemed to have a protective effect against benign prostatic hyperplasia and related symptoms in men. The associations with specific foods and tobacco use were inconsistent. Their use of a modified Oxford scale to grade the evidence revealed that the quality was generally low, with a majority at level 4, indicating a need for higher-quality research.

In comparison, our review specifically focused on dietary sensitivities and modifications in IC/BPS patients and observed that certain foods and drinks could exacerbate symptoms. The findings from Bradley et al. [38] are in harmony with ours regarding the impact of caffeine and fluid intake on urinary symptoms. However, while Bradley et al. [38] also found an inconsistency in the effects of specific foods, our review suggested a more direct link between food sensitivities and IC/BPS symptoms. This could be due to the broader scope of lower urinary tract symptoms examined by Bradley et al. [38], which are not exclusive to IC/BPS patients.

Colemeadow et al. [39] discussed the complexity of BPS etiology and the multimodal treatment approaches, emphasizing conservative treatment as the initial management strategy. This conservative approach includes patient education, behavioral modification, dietary advice, stress relief, and physical therapy. Their review suggests that behavioral and dietary changes are foundational in managing BPS and that these non-pharmacological interventions can significantly improve symptoms. This is consistent with our review’s findings that IC/BPS patients benefit from dietary interventions, such as the AID-IC diet and the ISDM approach. They often make spontaneous dietary changes in response to their symptomatology. Interestingly, both Bradley et al. [38] and Colemeadow et al. [39] mentioned the need for higher-quality evidence to understand the impact of these modifiable factors better. Our review also noted some divergences in methodology and specific dietary components, suggesting the need for more standardized research to clarify further the relationship between diet and IC/BPS symptoms.

Limitations

The collection of studies in this investigation has provided valuable insights, yet it is imperative to acknowledge the inherent limitations accompanying the findings. The studies, predominantly cross-sectional, give a snapshot of the association between diet and IC/BPS but are inherently limited in their ability to establish causality. Moreover, the reliance on self-reported data raises concerns regarding recall bias and the accuracy of dietary assessments. The heterogeneity of study designs, including differences in sample sizes, demographic characteristics, and dietary assessment tools, impedes the generalization of the results across the broader IC/BPS patient population. Because of this variability, the lack of standardized dietary interventions or definitions of ‘dietary modification’ further complicates the comparison of outcomes across studies.

Furthermore, the studies have primarily focused on exacerbating symptoms in response to specific dietary components without comprehensively understanding the underlying mechanisms. This gap in knowledge limits the development of targeted dietary recommendations and the experience of individual patient variability in dietary responses. The potential influence of psychosocial factors on symptom reporting was not consistently accounted for across studies, which may have introduced additional layers of complexity to interpreting the relationship between diet and symptomatology. Additionally, the limited duration of follow-up in interventional studies poses questions regarding the long-term sustainability and efficacy of dietary modifications. The presence of confounding factors such as smoking, as noted in the survey by Tettamanti et al. [23], and their potential interaction with dietary habits require careful consideration. Such confounders may obscure the proper relationship between diet and IC/BPS, necessitating more rigorous control in future research designs.

Clinical recommendations

The analyzed evidence recommends that individuals with IC/BPS actively examine their dietary habits to identify and minimize the intake of foods and beverages that may exacerbate their symptoms. Healthcare providers should consider integrating dietary counseling into the management plans for IC/BPS patients, focusing on avoiding known irritants such as certain acidic or spicy foods and possibly caffeine, as these have been implicated in symptom flare-ups.

Adopting structured dietary interventions, such as the AID-IC, may be beneficial and could potentially improve the quality of life for these patients. However, given the individual variability in dietary sensitivities, a personalized approach to diet modification is advisable. Patients might be encouraged to gradually adjust their diet based on self-observation and symptom tracking, starting with common irritants like coffee and exploring the effects of cranberry intake, as some patients have found these adjustments helpful. Using reliable and consistent tools to measure food sensitivities should also be incorporated into clinical practice to guide dietary recommendations better. Furthermore, the overall lifestyle of IC/BPS patients should be considered, with smoking cessation being a potential additional focus due to its association with bladder symptoms.

## Conclusions

After a thorough review and synthesis of the aggregate data, the studies revealed a discernible pattern, underscoring the influence of dietary factors on the clinical manifestation of IC/BPS. The evidence demonstrated a consistent link between the consumption of certain foods and beverages and the worsening of IC/BPS symptoms. Individuals with IC/BPS often reported heightened food sensitivities, which were correlated with increased severity of symptoms. In addition to structured dietary interventions, evidence suggests that IC/BPS patients may spontaneously adjust their nutritional habits in response to their symptoms. Such lifestyle and dietary changes appeared to be common coping mechanisms, indicating an intrinsic recognition of the diet-symptom relationship among patients.

Assessing the reliability of survey instruments used to measure food sensitivities added methodological rigor to the research, enhancing the credibility of the associations between diet and IC/BPS. Most studies focused on acidic and spicy foods, but some also examined caffeine and tea. These investigations highlighted the nuanced nature of dietary influences, showing that common irritants and seemingly benign substances could affect symptom management in IC/BPS.

## References

[1] Suskind AM, Berry SH, Ewing BA (2013). The prevalence and overlap of interstitial cystitis/bladder pain syndrome and chronic prostatitis/chronic pelvic pain syndrome in men: results of the RAND Interstitial Cystitis Epidemiology male study. J Urol.

[2] Hanno PM, Burks DA, Clemens JQ (2011). AUA guideline for the diagnosis and treatment of interstitial cystitis/bladder pain syndrome. J Urol.

[3] Shorter B, Ackerman M, Varvara M (2014). Statistical validation of the Shorter-Moldwin Food Sensitivity Questionnaire for patients with interstitial cystitis/bladder pain syndrome. J Urol.

[4] Suskind AM, Berry SH, Ewing BA, Elliott MN, Suttorp MJ, Clemens JQ (2013). The prevalence and overlap of interstitial cystitis/bladder pain syndrome and chronic prostatitis/chronic pelvic pain syndrome in men: results of the RAND Interstitial Cystitis Epidemiology male study. J Urol.

[5] McKernan LC, Walsh CG, Reynolds WS, Crofford LJ, Dmochowski RR, Williams DA (2018). Psychosocial co-morbidities in Interstitial Cystitis/Bladder Pain syndrome (IC/BPS): a systematic review. Neurourol Urodyn.

[6] Sutcliffe S, Bradley CS, Clemens JQ (2015). Urological chronic pelvic pain syndrome flares and their impact: qualitative analysis in the MAPP network. Int Urogynecol J.

[7] Akiyama Y (2021). Biomarkers in interstitial cystitis/bladder pain syndrome with and without hunner lesion: a review and future perspectives. Diagnostics (Basel).

[8] Jiang YH, Peng CH, Liu HT, Kuo HC (2013). Increased pro-inflammatory cytokines, C-reactive protein and nerve growth factor expressions in serum of patients with interstitial cystitis/bladder pain syndrome. PLoS One.

[9] Chung SD, Liu HT, Lin H, Kuo HC (2011). Elevation of serum c-reactive protein in patients with OAB and IC/BPS implies chronic inflammation in the urinary bladder. Neurourol Urodyn.

[10] Parsons CL, Argade S, Evans RJ (2020). Role of urinary cations in the etiology of interstitial cystitis: a multisite study. Int J Urol.

[11] Page MJ, McKenzie JE, Bossuyt PM (2021). The PRISMA 2020 statement: an updated guideline for reporting systematic reviews. BMJ.

[12] Sterne JA, Savović J, Page MJ (2019). RoB 2: a revised tool for assessing risk of bias in randomised trials. BMJ.

[13] Downes MJ, Brennan ML, Williams HC, Dean RS (2016). Development of a critical appraisal tool to assess the quality of cross-sectional studies (AXIS). BMJ Open.

[14] Sterne JA, Hernán MA, Reeves BC (2016). ROBINS-I: a tool for assessing risk of bias in non-randomised studies of interventions. BMJ.

[15] Balshem H, Helfand M, Schünemann HJ (2011). GRADE guidelines: 3. Rating the quality of evidence. J Clin Epidemiol.

[16] Bassaly R, Downes K, Hart S (2011). Dietary consumption triggers in interstitial cystitis/bladder pain syndrome patients. Female Pelvic Med Reconstr Surg.

[17] Gordon B, Blanton C, Ramsey R, Jeffery A, Richey L, Hulse R (2022). Anti-inflammatory diet for women with interstitial cystitis/bladder pain syndrome: the AID-IC pilot study. Methods Protoc.

[18] Jarman A, Janes JL, Shorter B (2023). Food sensitivities in a diverse nationwide cohort of veterans with interstitial cystitis/bladder pain syndrome. J Urol.

[19] Lin KB, Wu MP, Lin YK, Yen YC, Chuang YC, Chin HY (2021). Lifestyle and behavioral modifications made by patients with interstitial cystitis. Sci Rep.

[20] Oh-Oka H (2017). Clinical efficacy of 1-year intensive systematic dietary manipulation as complementary and alternative medicine therapies on female patients with interstitial cystitis/bladder pain syndrome. Urology.

[21] Shorter B, Ackerman M, Varvara M, Moldwin RM (2014). Statistical validation of the shorter-moldwin food sensitivity questionnaire for patients with interstitial cystitis/bladder pain syndrome. J Urol.

[22] Staack A, Distelberg B, Schlaifer A, Sabaté J (2017). Prospective study on the effects of regular and decaffeinated coffee on urinary symptoms in young and healthy volunteers. Neurourol Urodyn.

[23] Tettamanti G, Nyman-Iliadou A, Pedersen NL, Bellocco R, Milsom I, Altman D (2011). Influence of smoking, coffee, and tea consumption on bladder pain syndrome in female twins. Urology.

[24] Guedes PF, Santana SF, Colpo E (2019). Consumption of bladder irritant liquids and foods by incontinent women. Discip Sci.

[25] Geppetti P, Nassini R, Materazzi S, Benemei S (2008). The concept of neurogenic inflammation. BJU Int.

[26] (2024). Non-Neurogenic Female LUTS. https://uroweb.org/guidelines/non-neurogenic-female-luts.

[27] Chung E, Lee D, Gani J (2018). Position statement: a clinical approach to the management of adult non-neurogenic overactive bladder. Med J Aust.

[28] Takahashi S, Takei M, Asakura H (2021). Clinical guidelines for female lower urinary tract symptoms (second edition). Int J Urol.

[29] Elliott RA, Kapoor S, Tincello DG (2011). Expression and distribution of the sweet taste receptor isoforms T1R2 and T1R3 in human and rat bladders. J Urol.

[30] Dasgupta J, Elliott RA, Doshani A, Tincello DG (2006). Enhancement of rat bladder contraction by artificial sweeteners via increased extracellular Ca2+ influx. Toxicol Appl Pharmacol.

[31] Shahid M, Kim M, Yeon A, Andres AM, You S, Kim J (2018). Quantitative proteomic analysis reveals caffeine-perturbed proteomic profiles in normal bladder epithelial cells. Proteomics.

[32] Avelino A, Cruz F (2006). TRPV1 (vanilloid receptor) in the urinary tract: expression, function and clinical applications. Naunyn Schmiedebergs Arch Pharmacol.

[33] Dasgupta J, Elliott RA, Tincello DG (2009). Modification of rat detrusor muscle contraction by ascorbic acid and citric acid involving enhanced neurotransmitter release and Ca2+ influx. Neurourol Urodyn.

[34] Jordt SE, Bautista DM, Chuang HH (2004). Mustard oils and cannabinoids excite sensory nerve fibres through the TRP channel ANKTM1.. Nature.

[35] Verghese TS, Riordain RN, Champaneria R, Latthe PM (2016). Complementary therapies for bladder pain syndrome: a systematic review. Int Urogynecol J.

[36] Gordon B (2023). Nutritional considerations for bladder storage conditions in adult females. Int J Environ Res Public Health.

[37] Friedlander JI, Shorter B, Moldwin RM (2012). Diet and its role in interstitial cystitis/bladder pain syndrome (IC/BPS) and comorbid conditions. BJU Int.

[38] Bradley CS, Erickson BA, Messersmith EE (2017). Symptoms of Lower Urinary Tract Dysfunction Research Network (LURN). Evidence of the impact of diet, fluid intake, caffeine, alcohol and tobacco on lower urinary tract symptoms: A systematic review. J Urol.

[39] Colemeadow J, Sahai A, Malde S (2020). Clinical management of bladder pain syndrome/interstitial cystitis: a review on current recommendations and emerging treatment options. Res Rep Urol.

